# Agonism and performance in adolescent football players in informal physical education settings

**DOI:** 10.3389/fspor.2025.1511719

**Published:** 2025-01-30

**Authors:** Sara Aliberti, Francesca D’Elia, Giuseppe Giardullo, Gaetano Raiola

**Affiliations:** ^1^ Physical Education and Exercise Research Center, University Pegaso, Naples, Italy; ^2^ Department of Human, Philosophical, and Educational Sciences, University of Salerno, Baronissi, Italy; ^3^ Department of Neuroscience, Biomedicine and Movement, University of Verona, Verona, Italy

**Keywords:** assessment, emotional intelligence, soccer, monitoring, anxiety

## Abstract

Football is one of the most widely practiced sports in the world, and competition significantly influences athletic performance, especially in adolescents who experience emotional pressure that impacts their performance through the management of performance-related stress. Physical education is integrated within the school curriculum, but it is also typically delivered in sports associations engaged in competitive activities, which become informal learning environments as they pursue the same educational goals as schools. However, few studies have focused on this aspect, particularly the role of emotions and their relationship with performance anxiety in adolescent football players. The aim of this study was to analyze the relationship between pre-competitive anxiety and emotional regulation in adolescents regarding competitive performance. The study design was exploratory. A sample of 79 Under-19 football players, with a mean age of 14.6 (±1.89) years, was recruited through convenience sampling. A battery of pre-competition questionnaires was administered, including the Sport Anxiety Scale-2 (SAS-2) to measure anxiety levels and the Trait Emotional Intelligence Questionnaire (TEIQue-SF) to assess pre-competition emotional levels. Spearman's correlation was used to evaluate the strength and direction of the relationship between emotional levels and anxiety, while Chi-square test was employed to examine differences in anxiety levels across different player roles. The results showed that 70% of the football players displayed normal anxiety levels, with no significant differences across player positions. No significant correlation was found between emotional levels and anxiety. The primary causes of anxiety were cognitive concerns and bodily sensations, which negatively affected concentration. Although emotions were generally high, they did not appear to directly influence performance anxiety, suggesting that other factors may contribute to pre-competitive emotional regulation. In conclusion, contrary to common belief, performance anxiety in adolescent football competitions does not have a direct impact on emotional levels.

## 1 Introduction

Football is universally recognized as one of the most played and followed sports worldwide, capable of eliciting intense emotions among both players and fans. However, the interest in this sport extends beyond mere physical practice: numerous scientific studies have demonstrated that emotions play a crucial role in athletic performance, especially in high-pressure situations, such as those encountered in football competitions (1, 2). In addition to motor control, emotional regulation also plays a fundamental role in athletic performance, as emotions directly influence an athlete's ability to execute complex movements and manage pressure situations (3). Performance anxiety represents one of the main psychological constructs that affect an athlete's effectiveness during competition (4, 5). Adolescent sports performance is significantly impacted by emotional pressure, which influences performance through the management of performance-related stress. Physical education is framed within the school system but is also routinely practiced in sports associations that engage in competitive activities. These associations become informal learning environments because they pursue the same educational goals as schools (6, 7). However, few studies have focused on this aspect and on the role of emotions, particularly their relationship with performance anxiety in adolescent football players.

Performance anxiety arises when an athlete perceives competition as a highly significant event, often accompanied by a strong fear of failure or external judgment. This condition can lead the athlete to feel emotionally overwhelmed, resulting in debilitating effects on their ability to maintain focus and regulate physical effort. It is important to emphasize that anxiety is not inherently negative; rather, it is the ability to manage it that determines its positive or negative impact on sports performance (8). Proper management of anxiety and arousal can, in fact, transform these emotions into valuable resources, while the inability to regulate them may severely compromise performance (9). Emotional regulation and performance anxiety are thought to be intricately linked because the ability to manage one's emotions plays a crucial role in how an athlete perceives and responds to stressors. High levels of emotional intelligence (EI) may buffer against the detrimental effects of anxiety by enabling athletes to reframe stress-inducing situations, reduce excessive cognitive arousal, and maintain focus. Research indicates that athletes with higher EI are more effective in competitions, suggesting a direct and positive correlation between EI and sports performance (10). Additionally, good EI has been observed to regulate negative emotions during stressful situations, improving mental resilience and sports performance (11). Conversely, low EI could exacerbate the impact of anxiety by amplifying fears of failure or external judgment, leading to decreased concentration and impaired performance. This anticipated relationship underscores the importance of studying these variables in tandem to better understand their interaction and influence on adolescent athletes' performance.

EI, as defined by Salovey and Mayer (12), refers to the ability to perceive, understand, and regulate one's own emotions and those of others. This skill has proven to be particularly relevant in highly competitive contexts such as sports, where athletes are constantly exposed to stressful situations. EI enables athletes to modulate emotional responses during competition, conserving the mental and physical energy necessary for optimal performance. The relationship between EI and sporting success has been the focus of numerous studies, with evidence showing that effective emotional regulation can enhance not only competition performance but also the overall psychological well-being of athletes (13, 14). A striking example of how emotions can influence sports performance is provided by penalty kicks, one of the most analyzed situations in psychological literature on football. Jordet and Elferink-Gemser (15) studied the experiences of eight professional players who participated in the 2012 UEFA European Championship, observing that anxiety was the dominant emotion experienced by athletes before taking the kick. Emotional control in these moments is crucial: the ability to focus on the goal while excluding thoughts related to external judgment or fear of failure can make the difference between success and failure (16). An example of peak emotional concentration in this context can be seen in the behavior of Gianluigi Donnarumma after saving the decisive penalty in the Euro 2020 final. As explained by mental coach Nicoletta Romanazzi (17), the goalkeeper did not immediately realize he had won, as he was fully immersed in a state of “flow” and maximum concentration, diametrically opposed to the so-called “cocktail party” effect.

Despite the clear relevance of emotions in football, few studies have specifically focused on the role of EI and its relationship with performance anxiety within the educational processes commonly implemented in football academies. Adolescence represents a critical stage of development, during which emotional regulation abilities are not yet fully mature (32). This can lead to increased vulnerability to the negative effects of pre-game anxiety, with repercussions on both athletic performance and psychological well-being (18). Understanding how EI influences anxiety management during this stage can offer valuable insights for the development of training interventions and psychological support aimed at improving athletic performance and promoting the well-being of young athletes (19, 20). Furthermore, the ability to manage precompetitive anxiety does not only affect individual athletes, but it can also have a significant impact on the entire team. Recent research has highlighted that collective team emotions may have a greater influence on performance than the emotions of individual players (21). This emotional contagion phenomenon is particularly evident in team sports like football, where interaction and cohesion among teammates play a crucial role in achieving success. Studies suggest that higher EI not only helps athletes regulate their own emotions but also fosters a positive emotional climate within the team, enhancing cooperation and collective resilience (22, 23).

The aim of this study was to investigate the relationship between precompetitive anxiety and emotional regulation in adolescents in the context of athletic performance. The goal was to understand how these young athletes managed their emotions during sports competitions and to identify any potential gaps or strengths in this educational process. The analysis of the results will allow for the development of targeted intervention strategies aimed at improving athletes' emotional management while simultaneously promoting their psychological well-being and ability to successfully face sporting challenges. Exploring the link between performance anxiety and emotion, combined with the awareness of the importance of emotional regulation in youth football, represents a key objective in physical education within learning environments beyond the traditional school setting. This is a fundamental step toward understanding how to support and train adolescents—who are also athletes—to become resilient individuals capable of excelling in managing their emotions within the competitive sports experience. Such exploration not only enhances performance but also contributes to personal development, making sports competition a well-rounded educational experience.

## 2 Methods

### 2.1 Design and participants

This exploratory study recruited 79 Under-19 football players through convenience sampling, ranging in age from 12 to 19 years (mean, 14.6 ± 1.89 years old). Participants were sourced from youth football teams including Team 1 (23 players), Team 2 (20 players), Team 3 (14 players) and Team 4 (22 players). All participants were students, with 69.9% having played football for eight or more years. Among the players, 30% were midfielders, 29% defenders, 27% forwards, and 12% goalkeepers. The detailed demographic characteristics are shown in Table 1.

**Table 1:** Players role.

		*N*	%
Role	Forward	22	27.8
	Midfielder	24	30.4
	Defender	23	29.1
	Goalkeeper	10	12.7
	Total	79	100

The study was conducted in accordance with current ethical and regulatory guidelines for research with human subjects in the reference region. No formal ethics committee approval was required because the study was purely observational and did not involve intervention or treatment of participants. However, strict procedures were followed to ensure the protection of participants' rights and privacy. All data were collected anonymously and processed in accordance with local data protection regulations (GDPR). Additionally, written informed consent was obtained from all participants and, for minors, also from their legal guardians.

### 2.2 Data collection

The exploratory survey used paper-based questionnaires administered to young football players. The basic criteria for completing the questionnaire included active football practice and being aged between 12 and 19 years. Completion of the questionnaire took approximately 15 min, and the surveys were conducted on-site at the football clubs before training sessions. The objectives, tools used, and inclusion criteria were clearly explained to both the players and their sports directors, with an emphasis on the anonymous nature of the questionnaire. Before responding to the test questions, players were asked to read the informational document, and consent forms were signed by the parents of minors and autonomously by players of legal age. The questionnaire consisted of three sections: demographic information, the Sport Anxiety Scale-2 (SAS-2), and the Trait EI Questionnaire-Short Form (TEIQue-SF).

#### 2.2.1 Demographic information

Participants were asked to provide: **y**ear of birth, to satisfy inclusion criteria**, y**ears of experience in football, nationality, and position played (e.g., goalkeeper, midfielder, forward, etc.).

#### 2.2.2 Sport Anxiety Scale-2 (SAS-2)

The second section of the questionnaire utilized the Sport Anxiety Scale-2 (SAS-2), designed to assess sport-related performance anxiety experienced by athletes before or during competition (24, 33). The SAS-2 consists of 21 items with responses measured on a Likert scale, ranging from 1 (“almost never or rarely”) to 4 (“almost always”). The Italian version, translated by Dr. Massimo Masserini, was used in this study. The athletes completed the scale at their own pace, and it was used to assess three main factors: somatic anxiety, worry, and concentration disruption. Example items include: 1) “I feel more nervous and anxious than usual”; 8) “I feel tension in my stomach”; and 20) “I am afraid I will not be able to concentrate”. Scores are calculated by summing the responses to all 21 items, which fall into one of three categories: normal anxiety (21–40 points), moderate anxiety (41–60), and high anxiety (61–84). This test is particularly useful in research involving adolescents due to its brevity and ease of comprehension. The SAS-2 does not have absolute diagnostic value but can be used for self-assessment and to track changes over time.

#### 2.2.3 Trait EI Questionnaire (TEIQue-Sf)

The third section comprised the Trait EI Questionnaire-Short Form (TEIQue-SF), developed to assess trait EI according to the model of Petrides & Furnham (25, 26). Trait EI refers to various self-perceptions and dispositions related to emotions, situated at the lower levels of personality hierarchies, and is measured through self-report questionnaires. Petrides & Furnham's model identifies fifteen trait EI facets: adaptability, assertiveness, emotion expression, emotion management, emotion perception, emotion regulation, impulsiveness, relationship skills, self-esteem, self-motivation, social competence, stress management, trait empathy, trait happiness, and trait optimism. These facets form the basis of the TEIQue, which includes 153 items and covers four broad dimensions: Wellbeing, Self-control, Emotionality, and Sociability. For this study, the short form (TEIQue-SF) was used, comprising 21 items that sample the two best-saturating items for each of the fifteen sub-dimensions from the full version (27). This allows for a summary assessment of the trait EI levels among football players aged 12–19 years. The questionnaire employs a Likert scale ranging from 1 (“strongly disagree”) to 7 (“strongly agree”), and participants are encouraged to answer quickly and accurately without overthinking the exact meaning of the items.

### 2.3 Statistical analysis

Descriptive statistics were used to summarize the results in terms of means and standard deviations. The Chi-square test was employed to examine differences in anxiety levels across different player roles. Spearman's correlation was used to investigate relationships between variables such as age, anxiety levels (including its various forms), and EI levels. All data were analyzed using SPSS.27 software.

## 3 Results

Seventy percent of football players displayed a normal level of anxiety, scoring between 21 and 40 points. Only 29.1% of the players showed moderate levels of anxiety, with scores ranging from 41 to 60. Chi-square test revealed that the level of anxiety did not vary significantly according to the players' roles (*p* > 0.05). Table 2 shows the descriptive statistics for the items in the anxiety questionnaire.

**Table 2:** SAS2 – descriptive statistics.

Items	M	SD
Age	14.67	1.900
I feel more nervous and anxious than usual	2.01	.610
During competition, my thoughts wander elsewhere	1.65	.575
I have doubts about my abilities	1.78	.523
I feel my body is very tense and rigid	1.49	.552
I am worried about not expressing my full potential during the competition	2.30	.740
My mind wanders and is distracted during the competition	1.51	.596
During the competition, I am distracted by what is happening	1.67	.614
I feel tension in my stomach	1.84	.579
Negative thoughts influence my concentration	1.50	.552
I am worried about not having the right breath	1.20	.404
My heart beats too fast	2.01	.610
I feel my stomach is empty and/or heavy	1.07	.267
I am worried about performing poorly	2.30	.740
I lose concentration because I am nervous	1.72	.639
I feel shaky before or during the competition	1.24	.430
I am afraid of not achieving my goal	2.30	.740
My body feels compressed	1.49	.552
I am afraid of disappointing my coach and fans	1.98	.610
My stomach gets upset before or during a competitive event	1.84	.579
I am afraid I won't be able to concentrate	1.44	.500
My heart beats too fast before the competition	2.05	.552
Total score	36.48	7.248
Symptoms of worry	13.39	3.228
Concentration disruption	8.01	2.016
Somatic anxiety	15.07	3.312

The levels of EI averaged around 60 and did not vary significantly based on the players' roles. Table 3 presents the descriptive statistics for the items of the EI questionnaire.

**Table 3:** TEIQue-SF – descriptive statistics.

Items	M	SD
Expressing my emotions verbally is not a problem for me	2.58	.652
I often find it hard to see things from another person's perspective	2.32	.472
In general, I am an extremely motivated person	3.32	.472
I usually find it hard to regulate my emotions	1.32	.472
I generally don't find life enjoyable	1.00	.000
I am usually able to relate successfully with other people	3.26	.654
I tend to change my opinions frequently	1.69	.515
I often don't understand which emotion I am feeling	1.32	.472
I feel that I have many good qualities	3.62	.488
I often find it hard to fight for my rights	1.84	.735
I am usually able to influence other people's feelings	1.58	.652
In general, I have a pessimistic view of things	1.25	.437
People close to me often complain that I don't treat them well	1.00	.000
I often find it hard to regulate my life according to circumstances	1.15	.361
In general, I am able to handle stress	3.11	.679
I often find it hard to show my feelings to those close to me	1.29	.510
I can usually put myself in other people's shoes and feel their emotions	2.79	.822
I often find it hard to find the right motivation	1.37	.488
I am usually able to control my emotions when I want to	2.78	.592
In general, I am happy with my life	4.00	.000
I believe I have a lot of personal energy	3.56	.498
I tend to give in even when I know I’m right	1.88	.697
It seems that I have no control over other people's feelings	1.55	.499
In general, I believe things will go well for me in life	4.00	.000
I find it hard to connect with people, even those close to me	1.16	.373
Total IE level	60.75	2.81

From correlation analysis, several relationships between variables emerged:

• A strong positive correlation between the total score of the test and the symptoms of worry (*r* = 0.8), loss of concentration (*r* = .6), and level of somatic anxiety (*r* = .8). In other words, the variables that most significantly influence the increase in pre-competition anxiety are worries and physical sensations, followed by loss of concentration.
• A weak negative correlation between age and loss of concentration (*r* = −.2). As age increases, loss of concentration decreases.
• As pre-competition worries increase, loss of concentration also increases (*r* = .4), as well as the level of somatic anxiety (*r* = .5).
• As loss of concentration increases, cognitive anxiety also increases (*r* = .4).
• No correlation was found with EI. A detailed description is provided in Table 4.

**Table 4:** Correlations.

		1	2	3	4	5	6
1) Age	r	1					
	Sig.						
2) Worries symptom	r	.030	1				
	Sig.	.794					
3) Lost of concentration	r	-.245*	.483**	1			
	Sig.	.030	.000				
4) Somatic anxiety	r	-.098	.592**	.431**	1		
	Sig.	.390	.000	.000			
5) Total Anxiety	r	-.085	.868**	.687**	.853**	1	
	Sig.	.458	.000	.000	.000		
6) IE	r	-.165	-.050	.056	.038	-.012	1
	Sig.	.147	.659	.624	.742	.915	

*1-tail.

**2-tails.

## 4 Discussion

This study hypothesized that sports performance anxiety and emotional management, as assessed by EI, were negatively correlated. Indeed, it was assumed that a high level of EI could act as a protective factor against anxiety, facilitating more effective emotion regulation during competitive stress situations. However, the results did not confirm a significant relationship between these two constructs, suggesting that anxiety management might depend on other factors besides EI. The results of the performance anxiety data analysis revealed that most adolescent soccer players exhibited normal levels of anxiety (70.9%), while a minority (29.1%) demonstrated moderate anxiety levels. These findings aligned with existing literature, suggesting that performance anxiety was a common component of athletes’ experiences, particularly among young individuals still developing their emotional and cognitive skills. The homogeneous distribution of anxiety concerning playing roles indicates that performance anxiety was not influenced by the specific role played on the field. This result was particularly interesting as it contradicted the hypothesis that roles with greater responsibility, such as that of a goalkeeper or striker, may be associated with higher anxiety levels. This uniformity may be explained by the fact that, at the youth level, pressure and expectations were distributed evenly within the team, regardless of the role (28, 29). The levels of EI measured in the players showed an overall average of about 60 points, indicating that young players possess a good level of awareness and management of their emotions. However, in this case as well, no significant differences emerged based on the role played on the field, suggesting that EI is not necessarily related to the type of responsibility or pressure arising from the playing role (30). The lack of variation indicated that EI was a more stable aspect, less influenced by game dynamics compared to anxiety. Additionally, it may reflect the uniform training these athletes receive, both psychologically and technically.

The correlation analysis revealed that pre-competition worries, and physical sensations were the factors that most significantly influenced the increase in pre-competition anxiety. This correlation (*r* = .8) was highly significant, confirming the crucial role of cognitive and somatic components in the experience of performance anxiety. The strong relationship between total anxiety and loss of concentration (*r* = .6) suggested that anxiety negatively affected the ability to maintain attention during the game, compromising performance. The negative correlation between age and loss of concentration (*r* = −.2) indicated that as age increases, young players become slightly better able to maintain focus, consistent with the idea that experience and maturity help athletes better manage pressure and distractions during competition. A surprising result was the absence of a significant correlation between EI and anxiety levels, both somatic and cognitive. This observation could indicate that despite a good level of EI, young soccer players still experience performance anxiety, probably due to other factors not considered. For example, social pressure or external expectations could play a relevant role (31). In addition, the relative homogeneity of EI levels, combined with predominantly normal anxiety levels, could explain the absence of a significant relationship.

The findings provided valuable insights. First, it appears that performance anxiety, a widespread phenomenon among adolescent soccer players, is not significantly affected by playing role; furthermore, EI, while generally good, does not show a direct correlation with anxiety levels, suggesting that other factors may be at play in the management of pre-game emotions. The study has several limitations related to several factors, including the small sample size due to time constraints to administer the questionnaire to several teams. Specifically, the problems primarily concerned the timing of the distribution of the test, which occurred around mid-May, at the end of the football season. This meant that many players had already begun to miss practice due to the conclusion of the season, drastically reducing the number of available subjects. Secondly, a lack of knowledge and low interest on the part of teams in topics linking psychology to sports emerged. This underscores the importance of disseminating more knowledge of these topics within sports settings to ensure that they value the relationship between psychology and soccer. The requirement for consent for participation and data processing, while essential given the minors' age, lengthened data collection times and likely posed an additional obstacle to retrieving the data. Regarding the limitations of the questionnaire, one of the most significant was its duration. Although only a few questions were used for each of the four parts of the questionnaire, and the responses were meant to be quick (scoring numbers, selecting boxes, etc.), the attention span of the players decreased during the completion. Nonetheless, the questionnaire was manageable for all players aged between twelve and nineteen years. The fact that an anonymous questionnaire was designed reduced the effect of social desirability, but did not eliminate it, as peer pressure still exerts a certain influence on young players Furthermore, using predominantly closed-response questions did not allow for in-depth exploration of certain responses or choices. However, this research provides an indicative idea of the levels of sports performance anxiety, EI, and the correlations between these two constructs.

## 5 Conclusion

The study analyzed the relationship between sports performance anxiety and EI in adolescent soccer players, revealing that most participants exhibited normal anxiety levels, with no significant variations based on their playing roles. Despite young soccer players demonstrating a good level of EI, no direct correlation was found between EI and anxiety levels, suggesting that other factors may influence the management of pre-competition emotions. The results highlighted that cognitive worries and physical sensations are the primary contributors to increased pre-competitive anxiety, negatively affecting concentration and, consequently, athletic performance. Age appears to play a marginal role in reducing loss of concentration, likely due to the accumulation of experience. While the study presents several limitations, including a limited sample size and the need for further research to confirm the findings, it provides a useful foundation for future psychological and training interventions aimed at improving emotional management among athletes. Promoting greater awareness of the connection between emotions and sports performance could be crucial for developing resilient and psychologically prepared athletes capable of successfully facing sports challenges.

## Data Availability

The original contributions presented in the study are included in the article/Supplementary Material, further inquiries can be directed to the corresponding author.
